# Destabilization of the Dystrophin-Glycoprotein Complex without Functional Deficits in α-Dystrobrevin Null Muscle

**DOI:** 10.1371/journal.pone.0002604

**Published:** 2008-07-02

**Authors:** Tina M. Bunnell, Michele A. Jaeger, Daniel P. Fitzsimons, Kurt W. Prins, James M. Ervasti

**Affiliations:** 1 Department of Biochemistry, Molecular Biology and Biophysics, University of Minnesota, Minneapolis, Minnesota, United States of America; 2 Program in Cellular and Molecular Biology, University of Wisconsin, Madison, Wisconsin, United States of America; 3 Department of Physiology, University of Wisconsin, Madison, Wisconsin, United States of America; University of California, Berkeley, United States of America

## Abstract

α-Dystrobrevin is a component of the dystrophin-glycoprotein complex (DGC) and is thought to have both structural and signaling roles in skeletal muscle. Mice deficient for α-dystrobrevin (*adbn^−/−^*) exhibit extensive myofiber degeneration and neuromuscular junction abnormalities. However, the biochemical stability of the DGC and the functional performance of *adbn^−/−^* muscle have not been characterized. Here we show that the biochemical association between dystrophin and β-dystroglycan is compromised in *adbn^−/−^* skeletal muscle, suggesting that α-dystrobrevin plays a structural role in stabilizing the DGC. However, despite muscle cell death and DGC destabilization, costamere organization and physiological performance is normal in *adbn^−/−^* skeletal muscle. Our results demonstrate that myofiber degeneration alone does not cause functional deficits and suggests that more complex pathological factors contribute to the development of muscle weakness in muscular dystrophy.

## Introduction

The dystrophin-glycoprotein complex (DGC) is comprised of dystrophin, the dystroglycans (α and β), the sarcoglycans (α, β, γ and δ), sarcospan, the syntrophins (α1, β1, β2) and α-dystrobrevin [1]. In skeletal muscle, this large complex of membrane-associated proteins links the sub-sarcolemmal actin cytoskeleton to the extracellular matrix and is thought to provide protection from stresses imposed during muscle contraction [2]. Mutations in several DGC components result in muscular dystrophy in both humans and a variety of animal models. Dystrophin is one of the best characterized DGC proteins and its absence in humans and the *mdx* mouse leads to a destabilization of the DGC, sarcolemmal fragility, myofiber degeneration and muscle weakness [3]. The absence of functional sarcoglycans (α-β-γ-and δ-) or dystroglycan also result in muscular dystrophies with similar pathological features [4]–[6].

α-Dystrobrevin is a dystrophin-related protein that binds directly to dystrophin, syntrophin, and the sarcoglycan complex [7]–[10]. In addition, α-dystrobrevin binds to the intermediate filament proteins syncoilin and synemin, thereby linking the DGC to the intermediate filament network [11], [12]. The importance of α-dystrobrevin in maintaining healthy muscle is demonstrated by the α-dystrobrevin null (*adbn^−/−^*) mouse, which exhibits extensive myofiber degeneration [13]. However, several important questions remain regarding the function of α-dystrobrevin and the relationship of the *adbn^−/−^* muscle phenotype to other dystrophic animal models. In particular, whether the absence of α-dystrobrevin leads to DGC instability or causes deficits in muscle function remain to be assessed.

To further investigate the consequences of α-dystrobrevin deficiency, we examined the biochemical stability of the DGC and also measured the *in vivo* and *ex vivo* physiological performance of *adbn^−/−^* muscle. Our results suggest that the interaction between dystrophin and β-dystroglycan is compromised by the loss of α-dystrobrevin, implicating a role for α-dystrobrevin in stabilizing the DGC. However, muscle function was not compromised in *adbn^−/−^* mice, which demonstrates that substantial muscle cell necrosis can occur without adverse effect on physiological performance.

## Results

### α-dystrobrevin biochemically stabilizes the DGC

**Figure 1:**
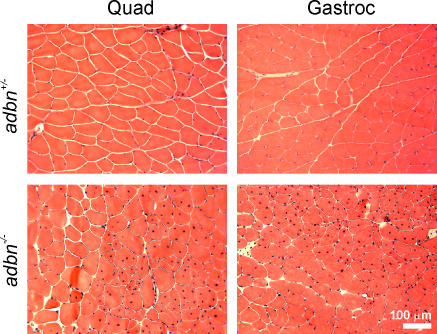
Histology of *adbn^+/−^* and *adbn^−/−^* muscle. Shown are representative hematoxylin and eosin stained sections of quadriceps and gastrocnemius muscle from 7 month *adbn^+/−^* and *adbn^−/−^* mice. Focal areas of centrally-nucleated fibers are evident throughout *adbn^−/−^* muscles, indicative of myofiber degeneration and regeneration.

We first assessed general histopathology by performing hematoxylin and eosin-phloxine staining on quadriceps, gastrocnemius and tibialis anterior muscles from 7 month old *adbn^+/−^* and *adbn^−/−^* mice (Fig. 1; tibialis anterior muscle not shown). Consistent with the findings of Grady et al. [13], *adbn^−/−^* skeletal muscle exhibited pockets of centrally-nucleated fibers, indicating that muscle degeneration and regeneration had occurred. Overall, approximately 50% of the fibers contained central nuclei in all muscle types examined. However, unlike other animal models in which DGC components have been ablated [14]–[20], we observed no evidence of fibrosis, mononuclear cell infiltration or adipose deposition.

**Figure 2:**
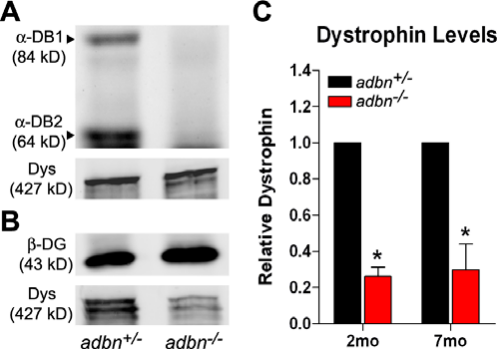
α-Dystrobrevin biochemically stabilizes the DGC. (A) Representative immunoblots for α-dystrobrevin and dystrophin from SDS-solubilized skeletal muscle. (B) Representative immunoblots for β-dystroglycan and dystrophin from digitonin-solubilized skeletal muscle enriched for the DGC using WGA-chromatography. (C) Quantification of the relative levels of dystrophin, after normalizing to β-dystroglycan intensity, from muscle solubilates incubated with WGA-agarose beads. Asterisks denotes a significant (one-sample t-test, p<0.05) reduction in the levels of dystrophin. n = 3 for each genotype/time point; error bars represent s.e.m.

Previous work implicated α-dystrobrevin as a linker protein between dystrophin and the sarcoglycan complex [10]. To assess whether α-dystrobrevin may play a structural role within the DGC, we measured the biochemical stability of the dystrophin-dystroglycan interaction in *adbn* ^−/−^ mice. Since it was initially reported that *adbn* ^−/−^ mice exhibit a progressive myopathy [13] we measured the biochemical stability of the DGC at both 2 and 7 months of age. Western blot analysis of SDS homogenates confirmed the absence of α-dystrobrevin in *adbn* ^−/−^ muscle (Fig. 2A). Total muscle lysates from *adbn* ^+/−^ and *adbn* ^−/−^ muscle also exhibited comparable levels of dystrophin (Fig. 2A) and β-dystroglycan (data not shown), consistent with reports from Grady et al. [21]. However, upon enrichment of the DGC from digitonin-solubilized muscle by wheat germ agglutinin (WGA) chromatography [22], dystrophin immunoreactivity in *adbn* ^−/−^ muscle was significantly reduced to 26% and 30% of that in *adbn^+/−^* muscle at 2 and 7 months of age, respectively (Fig. 2B and 2C). These data indicate that the biochemical interaction of dystrophin with the dystroglycan complex is compromised in the absence of α-dystrobrevin.

### Costameres are structurally normal in *adbn* ^−/−^ muscle

**Figure 3:**
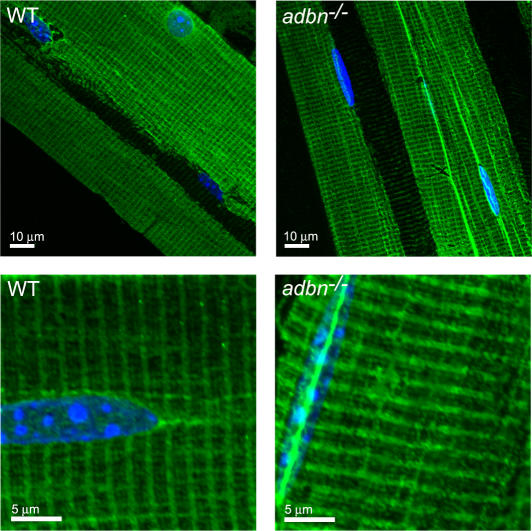
Normal costameres in *adbn^−/−^* muscle. Longitudinal cryosections of quadriceps from 7 month old mice stained with Rabbit 2 polyclonal antibody to dystrophin. Nuclei are stained blue.

It was previously shown that the dystrophin-deficient *mdx* mouse exhibits altered costamere organization [23], [24] and impaired costamere anchorage to the sarcolemma [25]. In addition to its association with dystrophin, α-dystrobrevin interacts both directly and indirectly with several intermediate filament proteins within the costameric network [11], [12]. Similar to the *mdx* mouse, mice deficient for desmin, an intermediate filament protein that interacts indirectly with α-dystrobrevin via its association with syncoilin [11], [26], display a loss of costamere structure in certain muscles [27]. Given that α-dystrobrevin interacts with proteins necessary for proper costamere formation and stabilizes the DGC (Fig. 2), we investigated costamere organization in *adbn^−/−^* mice. Both wild-type and *adbn^−/−^* skeletal muscle at 7 months of age exhibited highly organized rectilinear costamere structures with predominate dystrophin staining at the Z-line, finer transverse elements in register with the M-line and longitudinal elements (Fig. 3). These results indicate that α-dystrobrevin is not required for proper costamere organization.

### Physiological performance is normal in *adbn* ^−/−^ mice

To investigate the functional consequences of DGC instability (Fig. 2) and muscle cell death (Fig. 1) in *adbn* ^−/−^ mice, we assessed *ex vivo* and *in vivo* physiological performance using established techniques. Contractile properties of isolated extensor digitorum longus (EDL) muscles from *adbn* ^+/−^ and *adbn* ^−/−^ mice were determined at 2 and 7 months of age (Table 1). At both ages, isometric twitch force and specific force did not significantly differ between control and null muscle. Furthermore, *adbn^−/−^* muscle did not show an increased force drop after four eccentric contractions (ECCs). Since work done to stretch the muscle is the best predictor of the magnitude of contraction-induced injury [28], we calculated the combined work from all four ECCs and did not observe a significant difference between *adbn* ^+/−^ and *adbn* ^−/−^ muscle. In addition, we did not see an increase in Procion orange infiltration of *adbn^−/−^* myofibers compared to control after eccentric contractions (data not shown), indicating that sarcolemmal stability is not affected in *adbn^−/−^* muscle.

**Table 1:** Morphometric and contractile properties of EDL muscle.

Parameter	2 months		7 months	
	***adbn^+/−^***	***adbn^−/−^***	***adbn^+/−^***	***adbn^−/−^***
	n = 3	n = 3	n = 6	n = 6
*L_o_* (mm)	11.3±0.3	11.5±0.5	12.5±0.2	12.0±0.2
EDL mass (mg)	12.2±1.7	10.4±1.4	12.4±0.8	11.4±0.6
TPT (ms)	7.8±0.4	7.8±0.2	9.9±0.5	10.1±0.6
RT_1/2_ (ms)	8.3±1.8	11.3±3.1	13.1±0.9	12.5±0.7
Twitch force (mN)	22.0±4.0	28.6±7.9	45.0±5.1	34.1±4.0
Twitch force (mN/mm^2^)	10.4±2.7	14.5±2.8	22.2±3.8	16.7±1.7
Tetanic force (mN)	238.2±38.2	247.3±46.3	395.2±8.3	340.0±28.6
Tetanic force (mN/mm^2^)	111.4±27.3	127.1±12.7	190.1±15.8	166.1±8.6
Force drop (%)	24.1±4.3	33.0±8.6	20.5±1.8	22.0±4.8
Work (J/kg)	136.6±30.8	167.9±13.6	213.1±17.0	197.2±7.0

Results are presented as mean±s.e.m.

TPT, time to peak tension; RT_1/2_, half relaxation time

**Figure 4:**
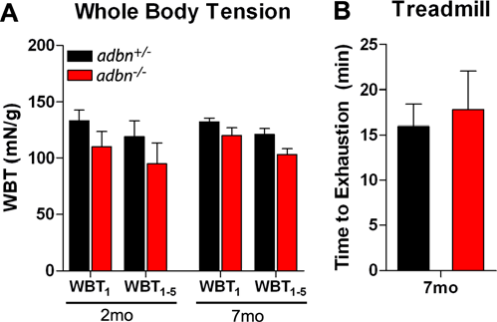
*adbn^−/−^* mice exhibit normal physiological performance *in vivo*. (A) Whole-body tension analysis depicting averages of the maximal response (WBT_1_) and the top five responses (WBT_1–5_) at 2 and 7 months of age. n≥3 for each genotype/time point. (B) Time to exhaustion during uphill treadmill running at 7 months of age. n = 4 for each genotype. No significant differences were observed. Error bars represent s.e.m.

Total body strength was assessed by measuring whole-body tension (WBT), defined as the forward pulling tension exerted by an individual mouse in response to a tail pinch stimulus [29]. Maximal responses (WBT_1_) and the average of the top five responses (WBT_1–5_) did not differ between *adbn* ^+/−^ and *adbn* ^−/−^ animals at either 2 or 7 months of age (Fig. 4A). To evaluate the endurance capacity of *adbn* ^−/−^ mice, maximal exercise performance tests were carried out using an uphill treadmill running regimen. At 7 months of age *adbn* ^−/−^ mice displayed similar times to exhaustion as *adbn* ^+/−^ mice (Fig. 4B). Together, these data demonstrate that despite biochemical destabilization of the DGC and substantial muscle necrosis, *adbn* ^−/−^ mice exhibit normal skeletal muscle function.

## Discussion

Since the initial observation that *adbn* ^−/−^ mice exhibit skeletal and cardiac muscle cell necrosis [13], the *adbn* ^−/−^ mouse has been considered an animal model for muscular dystrophy. However, as muscular dystrophy is characterized by both muscle cell death and muscle weakness, we examined the functional performance of *adbn^−/−^* muscle. Surprisingly, *adbn* ^−/−^ muscle was indistinguishable from control muscle using both *in vivo* and *ex vivo* analyses. These results were unanticipated, as *adbn^−/−^* mice exhibit an elevated presence of centrally-nucleated fibers similar to other mouse models of muscular dystrophy [13]. Although the exact cause(s) of muscle cell death and weakness in muscular dystrophy remains to be determined, it is reasonable to postulate that cell death contributes to the development of muscle weakness. Our results suggest that a more complex pathomechanism underlies the decreased physiological performance associated with muscular dystrophies.

Some fundamental differences in the histological observations between *adbn* ^−/−^ mice and other DGC-associated muscular dystrophy mouse models may help to explain the lack of a functional deficit in *adbn* ^−/−^ mice. Unlike muscle from dystrophin-, sarcoglycan- and dystroglycan-deficient animals [14]–[20], *adbn^−/−^* muscle does not exhibit significant fibrosis, mononuclear cell infiltration or increased membrane damage. Therefore, fibrosis, inflammation and membrane fragility may be factors contributing to the force deficits observed in dystrophic animal models. In fact, replacement of muscle fibers with less elastic fibrotic connective tissue has previously been suggested to contribute to decreased force production [30]. Furthermore, treatment of dystrophic mice and humans with corticosteroids, which is believed to help reduce inflammation, improves muscle function [31], [32], supporting the idea that inflammation also contributes to muscle weakness.

While the cause of muscle cell death in DGC-related muscular dystrophies is unclear, a leading hypothesis is that damage to the sarcolemma during muscle contraction causes a loss of calcium homeostasis, leading to activation of proteases and eventual cell death [33]. However, similar to our findings in *adbn^−/−^* mice, Warner et al. [34] report that Dp260/*mdx* mice exhibit muscle cell necrosis in the absence of sarcolemmal damage. These results indicate that sarcolemmal instability is not the sole cause of cell death. In the absence of sarcolemmal fragility, it has been hypothesized that cell death in *adbn* ^−/−^ mice results from aberrant signaling. Grady et al. [13] demonstrated a loss of sarcolemmal-localized neuronal nitric oxide synthase (nNOS) and impaired nNOS signaling in *adbn* ^−/−^ mice [13]. Impaired nNOS signaling leads to vasoconstriction and thereby decreased blood flow in active muscle [35]. Such ischemic conditions could explain the focal areas of necrosis observed in *adbn* ^−/−^ mice, which is similar to the histology of δ-sarcoglycan null mice in which vascular function is perturbed [36]. However, the loss of nNOS alone is unlikely to account for the dystrophy in *adbn* ^−/−^ mice since nNOS-deficient mice are not detectably dystrophic [37]. The question therefore remains as to what causes cell death in the absence of sarcolemmal damage. Understanding the mechanism underlying muscle degeneration in *adbn* ^−/−^ mice may provide insight into the molecular and cellular requirements for muscle cell death in other animal models of muscular dystrophy.

While it has previously been proposed that α-dystrobrevin plays a structural role in the DGC [10]–[12], we provide the first direct evidence that α-dystrobrevin helps to biochemically stabilize the interaction of dystrophin with the dystroglycan complex. Prior immunohistochemical analyses in *adbn^−/−^* mice have suggested that all other DGC components are expressed at normal levels and localize properly to the sarcolemma [13]. Similarly, we found that total dystrophin levels were equivalent in control and *adbn^−/−^* skeletal muscle extracts, but reduced by 70% in DGC-enriched fractions from *adbn^−/−^* muscle. This result suggests that the association between dystrophin and the glycoprotein complex is compromised in the absence of α-dystrobrevin. Although the destabilization of the DGC may contribute to the muscle cell necrosis in *adbn^−/−^* mice, it appears that the degree of dystrophin destabilization is insufficient to render the membrane susceptible to damage, cause costamere disorganization or alter the functional performance of the skeletal muscle. Furthermore, the upregulation of the cytoskeletal protein syncoilin [38] may be providing compensatory structural stabilization to the sarcolemma in the absence of α-dystrobrevin.

In addition to the sarcolemma, α-dystrobrevin is also enriched at the neuromuscular junction (NMJ). Previous work has shown that *adbn* ^−/−^ mice exhibit defects in the maturation and maintenance of the postsynaptic membrane at the NMJ as the result of increased mobility and turnover of acetylcholine receptors (AChRs) [21], [39]. Similarly, dystroglycan deficiency also disrupts the organization and stability of AChR clusters [40]. These studies suggest that the DGC is important for anchoring AChRs at synapses and maintaining postsynaptic receptor density at the NMJ. Our data illustrating the instability of the DGC in *adbn* ^−/−^ mice provides a possible explanation for the increased AChR mobility. While acetylcholinesterase (AChE) mobility is also increased in *adbn* ^−/−^ mice [41], it is unclear whether this is an indirect result of changes in AChRs or whether α-dystrobrevin and the DGC play a direct role in AChE stability. Nonetheless, disruption of AChE stability could result in prolonged muscle hyperexcitability, which over time may contribute to the observed cell death in *adbn* ^−/−^ mice. In support of this idea, mutations in the *Caenorhabditis elegans* dystrobrevin-like gene cause hypercontractility, which leads to muscle degeneration when on a sensitized background [42].

Study of the *adbn* ^−/−^ mouse has provided insight into the pathological factors that contribute to the development of muscular dystrophy. The observation that muscle cell necrosis and functional deficits are separable phenotypes forces us to reconsider the traditional view that muscle cell death is a causative factor for muscle weakness in muscle diseases. Furthermore, the *adbn* ^−/−^ mouse suggests that membrane fragility is not the sole contributing factor for muscle cell death in DGC-related muscular dystrophies. Further studies are needed to compare the cellular and molecular differences between the *adbn* ^−/−^ mouse and other DGC-related mouse models in order to help dissect the mechanisms underlying the development of muscular dystrophies.

## Materials and Methods

### Animals

A breeding pair of *adbn^−/−^* mice was generously provided by Dr. R. Mark Grady (Washington University, St. Louis, MO). To generate *adbn^+/−^* mice for controls, we outcrossed an *adbn^−/−^* male (SV129J/ C57Bl/6J mixed background) to an SV129J wild-type female to obtain *adbn^+/−^* mice. Subsequent mating between *adbn^+/−^* and *adbn^−/−^* mice provided *adbn^−/−^* and littermate control *adbn^+/−^* animals. Genotypic analyses were performed by standard PCR methods.

### Light and Confocal Microscopy

For light microscopy, muscles were dissected from *adbn^−/−^* and *adbn^+/−^* 7 month-old mice, quickly frozen in liquid nitrogen-cooled isopentane and mounted in O.C.T medium (TissueTek, Torrance, CA). 10 µm cryosections were cut on a Leica CM3050 cryostat, air-dried and stained with hematoxylin and eosin-phloxine. Images were obtained on a Zeiss Axiovert 25 microscope fitted with a Leica DFC300 FX camera using Image Pro Plus 5.1 software.

Costamere images were obtained according to previously described methods [23]. Mice were trans-cardially perfused with ice-cold 2% paraformaldehyde in phosphate buffered saline (PBS). Quadriceps were dissected, adhered to a cryostat chuck with O.C.T. medium and quickly frozen in liquid-nitrogen slush. 20 µm longitudinal cryosections were cut and stored at -80°C. For confocal microscopy, sections were thawed, blocked for 30 minutes in PBS/BSA (PBS containing 1 mg/ml bovine serum albumin and 10 mM NaN_3_) and incubated with a 1∶100 dilution of Rabbit 2 polyclonal antibody to dystrophin [25] for 2 hours at room temperature. Sections were rinsed with PBS/BSA, incubated with 1∶200 Alexa-488-conjugated 2° antibody (Molecular Probes, Carlsbad, CA) for 30 minutes at room temperature and rinsed with PBS/BSA. To visualize nuclei, 8 µM TO-PRO 3 iodide (Molecular Probes, Carlsbad, CA) was applied to sections for 5 minutes, rinsed with PBS/BSA and a coverslip applied with a drop of Slow-Fade Reagent (Molecular Probes, Carlsbad, CA). Slides were viewed on an Olympus Fluoview 1000 inverted confocal microscope with a 60x (NA 1.4) oil objective at the University of Minnesota-Twin Cities Biomedical Imaging Processing Laboratory. Laser power and PMT voltage were adjusted so that 2° antibody alone did not produce signal.

### Biochemical Analysis of DGC Stability

Skeletal muscle was dissected, snap-frozen in liquid nitrogen, and pulverized in a liquid nitrogen-cooled mortar and pestle. Total muscle homogenates were prepared by solubilizing pulverized muscle in 1% SDS, 5 mM EGTA, and protease inhibitors for 2 minutes at 100°C. The supernatant was collected after brief centrifugation and its protein concentration determined using the Bio-Rad *DC* Protein Assay Kit II (Bio-Rad, Hercules, CA). To enrich for the DGC, pulverized muscle was solubilized in 10 volumes/g of 50 mM Tris-HCl, 0.5 M NaCl, 1% digitonin and a cocktail of protease inhibitors for 1 hour at 4°C with gentle mixing. After high-speed centrifugation, the supernatant was applied to a WGA-Sepharose column (Sigma-Aldrich, St. Louis, MO) pre-equilibrated with wash buffer (50 mM Tris-HCl, 0.5 M NaCl, 0.1% digitonin, and protease inhibitors) for 2 hours at 4°C to enrich for dystroglycan and associated proteins. The column was washed and bound proteins eluted with Laemmli sample buffer (3% SDS and 1% β-mercaptoethanol) at 100°C. Dystrophin and β-dystroglycan immunoreactivity in SDS muscle solubilates (50 µg total protein) and WGA elutes were measured by quantitative western blot analysis using the monoclonal antibodies NCL-DYS1 to dystrophin and NCL-b-DG to β-dystroglycan (Novocastra Laboratories Ltd, Newcastle upon Tyne, UK). Secondary antibodies were IRDye 800- or IRDye680-conjugated goat anti-mouse IgG and the fluorescent signals were detected and quantified using the Odyssey Infrared Imaging System (LI-COR Biosciences, Lincoln, NE). Relative levels of dystrophin from WGA elutes were determined by normalizing dystrophin signals to the corresponding β-dystroglycan signal. Statistical significance at p<0.05 was determined using a one-sample t-test.

### *Ex Vivo* Force Measurements

All force measurements were obtained as previously described [43] from both male and female mice at 2 and 7 months of age. Experiments were conducted on both the right and left EDL muscle for each mouse. The EDL muscle was dissected and one tendon attached to a rigid support and the other to a dual-mode servomotor. The muscle was allowed to equilibrate in a Ca^2+^-Ringer's solution continuously gassed with 95% O_2_/5% CO_2_ to maintain a pH of 7.6 at 30°C. Platinum plate electrodes were positioned on either side of the muscle and stimulation induced by a single pulse lasting 200 microseconds. The muscle was adjusted to the optimal length (*L_0_*) at which maximal twitch force was achieved. Muscle was then subjected to an ECC regimen which consisted of five maximal tetanic stimulations at a frequency of 150 Hertz. Each stimulation was carried out over 700 milliseconds; over the final 200 milliseconds the muscle was lengthened at a velocity of 0.5 *L_0_*/second resulting in a total stretch of 10% *L_0_*. A five-minute recovery time was allowed between measurements. The average force produced during a stretch was calculated by integrating the force-time curve and dividing this value by the duration of the stretch. Work was calculated by multiplying the average force produced during a stretch by the length of the displacement and normalized to muscle mass. Upon completion of the ECC protocol, muscle was weighed and subsequently bathed in 0.1% Procion orange, washed, and frozen for cryosectioning. Cross sectional area of each muscle was calculated according to Brooks and Faulkner [44] by dividing muscle wet mass by the product of *L_0_*, the EDL fiber-to-muscle length constant 0.44, and 1.06 grams/centimeter^3^, the density of mammalian skeletal muscle [44]. Differences between genotypes at each time point were assessed by using a Student's two-tailed t-test for independent samples to determine significance at p<0.05.

### *In Vivo* Physiological Performance

The WBT protocol was carried out as previously described [43]. A mouse was attached by the tail to a horizontally mounted force transducer using suture silk and subsequently placed in an apparatus that allowed only forward movement. The force evoked by gentle tail pinches (approximately 5 pinches per minute) was recorded for 5 minutes and the top 5 values were identified and normalized to body mass.

Maximal exercise performance was tested on a Columbus Instruments treadmill with an uphill grade of 15°. Mice were acclimated to the treadmill by running at a speed of 10 meters per minute for 5 minutes, three times a week, for two weeks. To determine maximal exercise performance mice were run on the treadmill for 5 minutes, at a speed of 10 meters per minute, followed by a 1 meter per minute increase in speed every minute until exhaustion. Mice were considered exhausted when they refused to stay off a shock bar for at least 5 seconds. Maximal exercise capacity was determined as the average duration of two trials separated by two days. Differences between *adbn^+/−^* and *adbn^−/−^* mice at each time point were assessed by using a Student's two-tailed t-test for independent samples to determine significance at p<0.05.
